# Comment on the CUSUM surgical learning curve analysis in Dimitrovska *et al.* (2022)

**DOI:** 10.1093/icvts/ivac184

**Published:** 2022-07-25

**Authors:** George Rakovich, William H Woodall, Stefan Steiner

**Affiliations:** Section for Thoracic Surgery, Department of Surgery, Maisonneuve-Rosemont Hospital, University of Montreal School of Medicine, Montreal, QC, Canada; Department of Statistics, Virginia Tech, Blacksburg, VA, USA; Department of Statistics and Actuarial Sciences, University of Waterloo, Waterloo, ON, Canada

The cumulative sum (CUSUM) analysis included in the recent paper by Dimitrovska *et al.* [1] illustrates the major pitfall of using this popular approach to reach conclusions about the surgical learning process. Below we refer to the time series plot of operation times (Figure 1 [1]) and the corresponding CUSUM plot (Figure 2 [1]) from their paper [1].

**Figure 1:**
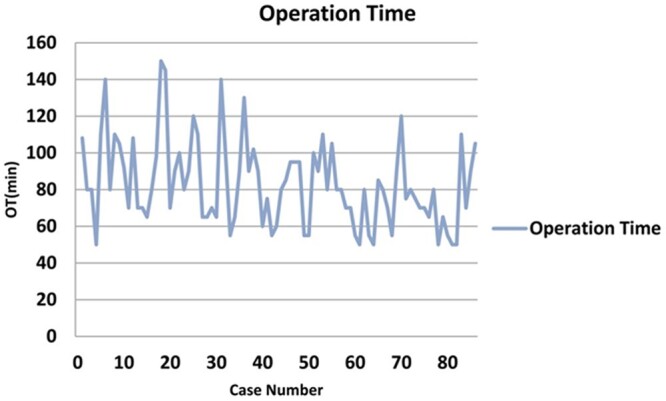
Time series plot of operating times. (From: Dimitrovska NT, Bao F, Yuan P, Hu S, Chu X, Li W. Learning curve for two-port video-assisted thoracoscopic surgery lung segmentectomy. Interact CardioVasc Thorac Surg 2021; Figure 3[1]).

The CUSUM plot of the sum of the successive differences of the operating times from their average (Figure 2 [1]) was broken into 3 phases, i.e. the initial learning, the increased competence and the experienced phases. As pointed out by Woodall *et al.* [2], however, this type of plot is subject to over-interpretation. It has neither a conceptual nor a mathematical justification.

**Figure 2:**
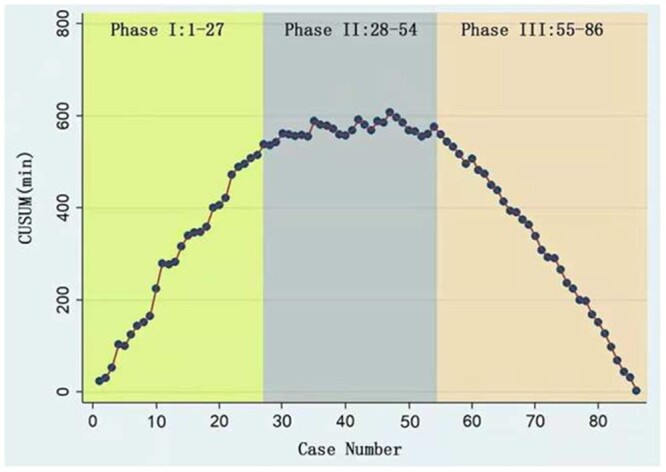
Cumulative Sum (CUSUM) plot of the successive differences of operating times from their average. (From: Dimitrovska NT, Bao F, Yuan P, Hu S, Chu X, Li W. Learning curve for two-port video-assisted thoracoscopic surgery lung segmentectomy. Interact CardioVasc Thorac Surg 2021; Figure 4[1]).

One can see from the time series plot in Figure 1 [1] that the average operation times are decreasing roughly linearly over time. By definition, any decreasing pattern in operation times will result in a parabolic-type pattern [2] such as that in Figure 2 [1]. As a result, the classification and interpretation of the 3 phases given in Dimitrovska *et al.* [1] are not justified. In addition, it should be noted that adding any constant to the operation times, such as an additional 30 min, would have absolutely no effect on the CUSUM plot in Figure 2 [1].

We believe that a far better approach would be to fit a statistical model to the raw operation time data and interpret that model. In this particular case, it seems that a simple linear regression model would fit the data fairly well. It is not clear from Figure 1 [1] whether further decreases in operation time could occur for future cases or if stability in operation times has been reached.

**Conflict of interest:** George Rakovich receives speaker fees from Medtronic^®^ and a research grant from Baxter^®^.

## Notes

The manuscript has not previously been published in print or electronic form and is not under consideration by another publication.
